# Transcriptome Sequence and Plasmid Copy Number Analysis of the Brewery Isolate *Pediococcus* *claussenii* ATCC BAA-344^T^ during Growth in Beer

**DOI:** 10.1371/journal.pone.0073627

**Published:** 2013-09-06

**Authors:** Vanessa Pittet, Trevor G. Phister, Barry Ziola

**Affiliations:** 1 Department of Pathology and Laboratory Medicine, University of Saskatchewan, Saskatoon, Saskatchewan, Canada; 2 Department of Food, Bioprocessing, and Nutrition Sciences, North Carolina State University, Raleigh, North Carolina, United States of America; University Medical Center Utrecht, Netherlands

## Abstract

Growth of specific lactic acid bacteria in beer leads to spoiled product and economic loss for the brewing industry. Microbial growth is typically inhibited by the combined stresses found in beer (e.g., ethanol, hops, low pH, minimal nutrients); however, certain bacteria have adapted to grow in this harsh environment. Considering little is known about the mechanisms used by bacteria to grow in and spoil beer, transcriptome sequencing was performed on a variant of the beer-spoilage organism *Pediococcus* *claussenii* ATCC BAA-344^T^ (Pc344-358). Illumina sequencing was used to compare the transcript levels in Pc344-358 growing mid-exponentially in beer to those in nutrient-rich MRS broth. Various operons demonstrated high gene expression in beer, several of which are involved in nutrient acquisition and overcoming the inhibitory effects of hop compounds. As well, genes functioning in cell membrane modification and biosynthesis demonstrated significantly higher transcript levels in Pc344-358 growing in beer. Three plasmids had the majority of their genes showing increased transcript levels in beer, whereas the two cryptic plasmids showed slightly decreased gene expression. Follow-up analysis of plasmid copy number in both growth environments revealed similar trends, where more copies of the three non-cryptic plasmids were found in Pc344-358 growing in beer. Transcriptome sequencing also enabled the addition of several genes to the *P* *. claussenii* ATCC BAA-344^T^ genome annotation, some of which are putatively transcribed as non-coding RNAs. The sequencing results not only provide the first transcriptome description of a beer-spoilage organism while growing in beer, but they also highlight several targets for future exploration, including genes that may have a role in the general stress response of lactic acid bacteria.

## Introduction

Beer is a challenging environment for microbes, as most of the nutrients have been utilized by yeast during fermentation. Furthermore, low pH and oxygen content, high CO_2_, and the presence of the antimicrobial compounds hops and ethanol leads to inhibition of most microbial growth. Nevertheless, certain organisms have adapted to overcome these stresses and thus can grow in beer. These microbes are typically not harmful if consumed; their growth, however, does produce off-flavours and turbidity, which spoils the product and leads to economic losses for the brewing industry.

The most common beer-spoilage organisms (BSOs) are lactic acid bacteria (LAB), in particular, those classified as *Lactobacillus* and *Pediococcus* spp. An interesting trait of these BSOs is that the ability to spoil beer is not uniform, as different strains of the same species may or may not be able to grow in beer. This leads to the difficult problem of BSO detection in breweries, as classic species-specific methods cannot indicate beer-spoilage potential. Prediction of whether an isolate can grow in beer is needed for accurate assessment of brewery contaminants. While growth-based assays can provide a good evaluation of spoilage potential, they are time-consuming and lead to stagnancy in the production cycle. To overcome these constraints, recent research has focused on molecular approaches to deliver quick and effective microbial quality control. A handful of genes have been proposed as markers for beer-spoilers, however, they are typically only 80-85% accurate [1]. This is partially due to genetic variation among BSOs, but also because some isolates have no potential beer-spoilage-related (BSR) genes (such as *hitA*, *horA*, and *horC*), yet are still able to grow in beer. As such, brewing microbial quality control research needs to broaden the understanding of how these isolates are able to adapt and grow in beer, particularly if more robust BSO detection methods are to be developed. Further, by studying LAB in the unique environment of beer, insight in to how these organisms adapt to survive in harsh environments should provide information that is applicable to other industries that involve LAB in stressful niches (e.g., food and fuel alcohol fermentations).

One of the recently preferred methods for analyzing organisms in an environment is global transcriptome sequencing, or RNA-seq. Originally developed for eukaryotes, the method uses high-throughput sequencing to capture a snapshot of RNA transcripts in the cell [2]. Gene expression levels can then be compared to assess the transcriptional response to different environments, similar to what has been done in the past via microarrays. Unlike microarrays, however, no prior knowledge of the organism is needed to perform RNA-seq (i.e., a genome sequence or annotation is not required). Typically, transcriptome sequencing techniques involve poly-A tail selection for preferential sequencing of cDNA derived from eukaryotic mRNA; however, this technique does not work for prokaryotes. Instead, ribosomal RNA (rRNA) must be removed before library preparation and sequencing, as it composes the majority of total RNA in bacterial cells. RNA is then fragmented, reverse-transcribed into cDNA, and sequenced with high-throughput technology. The sequencing reads can then be mapped back to the genome (if available) to get a global view of gene expression levels in a given environment. This RNA-seq technique has increasingly been used for both prokaryotic and eukaryotic organisms growing in a variety of environments (e.g., *Saccharomyces cerevisiae* [3], *Neisseria* [4], *Campylobacter* [5], and the LAB *Lactobacillus plantarum* [6]).

One beer-spoilage organism of particular interest is the brewery isolate *Pediococcus* *claussenii* ATCC BAA-344^T^ (Pc344) whose genome was recently sequenced [7]. Originally described in 2002 [8], this species is one of the more commonly found BSOs. Pc344 has the ability to spoil beer in 6-7 days (mid-range for BSOs) [9], and has moderately high hop resistance (unpublished data). The original brewery isolate has a genome of just over 1.8 Mbp, and eight plasmids. Two of the plasmids are small and cryptic, whereas the other six range from 16–36 kb and contribute roughly 7% of Pc344’s coding capacity [7]. Plasmid pPECL-8 contains the gene *horA* [10], however, no other putative BSR genes (e.g., *hitA, horC* [11,12]) are found in this isolate. RNA extraction from the original Pc344 brewery isolate proved to be very difficult, as it has the ability to produce a thick exopolysaccharide (“rope”). As such, a variant which had lost the glucosyltransferase gene *gtf* (found on plasmid pPECL-7) was used, since no phenotypic difference in growth in beer or ethanol/hop tolerance was found between the ropy (Pc344) and non-ropy (Pc344NR) isolates [13,14]. It was later noted after transcriptome sequencing that two additional plasmids (pPECL-4 and pPECL-6) had been lost during growth of the non-ropy isolate in preparation for sequencing. pPECL-4 and pPECL-6 do not appear to be required for Pc344 to grow in beer, as the variant missing these two plasmids (designated here as Pc344-358) was phenotypically similar to Pc344NR. Thus, we describe here the transcriptional response of Pc344-358 during growth in the contrasting environments of beer and MRS broth (MRS-B), with the goal of broadening our understanding of genes that are important for LAB growth in beer.

## Results and Discussion

### Sequencing and mapping

**Figure 1:**
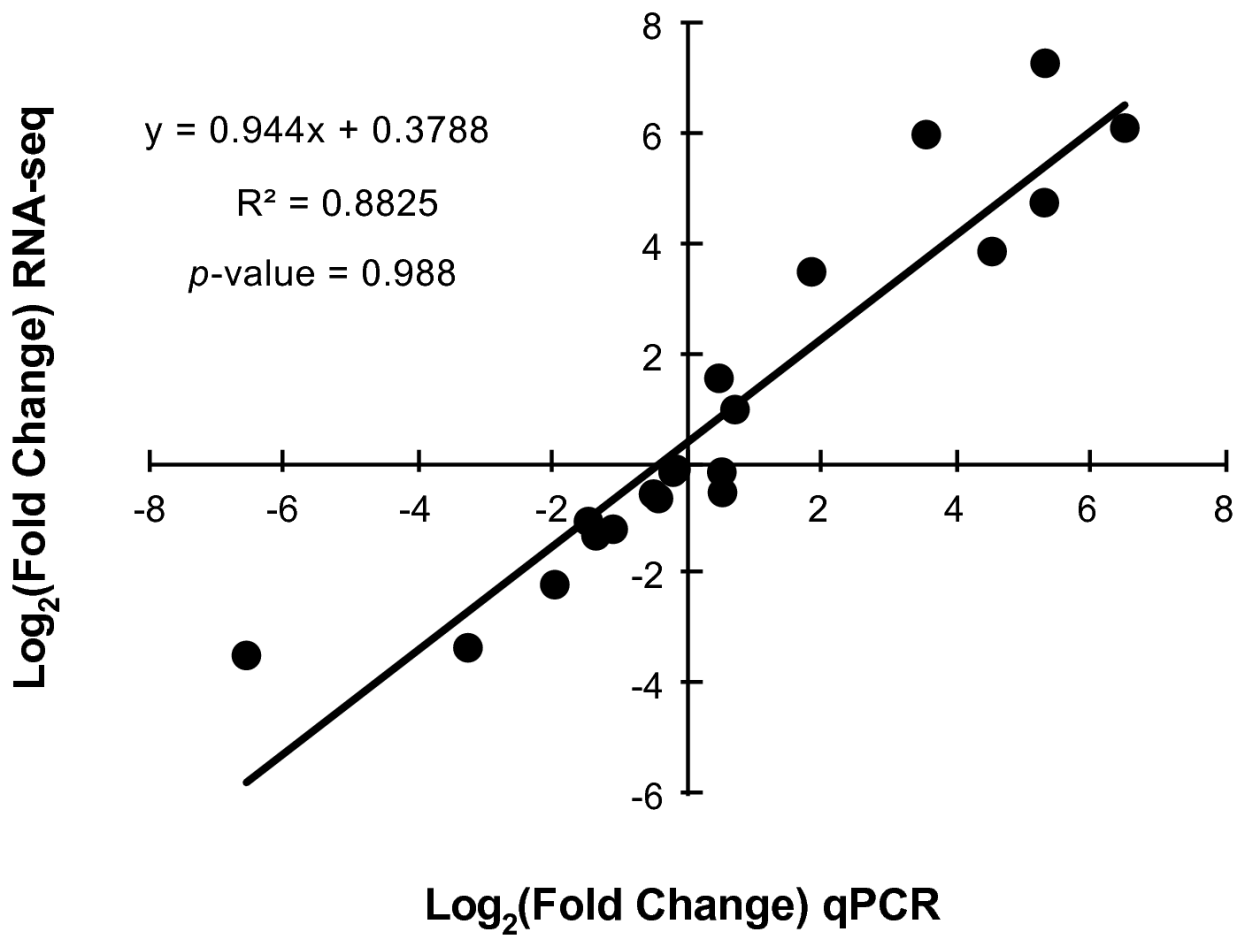
Comparing gene expression results from RT-qPCR and RNA-seq. Differential expression of twenty genes was analyzed by RT-qPCR and calculated log_2_ fold change in expression was plotted against the results obtained with transcriptome sequencing (statistical goodness of fit value is provided).

RNA was extracted from two biological replicates of Pc344-358 growing in MRS-B, a common LAB laboratory medium, and in beer. Transcriptome sequencing in a single lane of the Illumina platform provided 19-32 million reads for each sample (Table 1). No difference in expression results was found when raw or quality-filtered reads were aligned to the genome (data not shown). We therefore used unfiltered libraries as they provided better coverage of the Pc344 genome for annotation verification. Approximately 86% of the unfiltered reads aligned to the Pc344 genome, with 95% found in annotated open reading frames (ORFs; Table 1). Initially, however, only 75% of the beer sample reads were aligned in ORFs (not including rRNA or tRNA reads), whereas 91% of reads from the MRS-B samples represented annotated genes. All transcriptome sequencing data was therefore used to verify the Pc344 genome annotation and 21 genes were added, with half being preferentially transcribed during growth in beer (Table S2). rRNA removal via the MICROB*Express* kit only partially worked for the beer samples, where 64-78% of reads represented rRNA (Table 1). To overcome the differences in rRNA removal efficiency, reads aligning to 5S, 16S, or 23S rRNA genes from each library were not included in downstream analyses. Reads aligning to tRNA genes were also removed, as their small size (~70 nt) can lead to biases during library preparation. To compare transcript level results from each biological replicate, the reads per kilobase per million reads sequenced (RPKM) [2] were calculated to account for differences in library size. Excellent correlation was found between biological replicate RPKM values, with R^2^ values of 0.979 and 0.986 for beer and MRS-B samples, respectively (Figure S1). In addition, a comparison was made between transcriptome sequencing and reverse transcription quantitative PCR (RT-qPCR) techniques. Nine genes were chosen for RT-qPCR analysis based on varying gene lengths and differences in transcript levels, with five showing increased levels in beer, two having increased levels in MRS-B, and two showing no significant difference (Table S1). Results from a previously published transcriptional analysis of eleven genes [15] were also included for RNA-seq verification, as all RT-qPCR assays were performed at the same time on the same cDNA stocks. A good correlation was found between RNA-seq and RT-qPCR results for the twenty genes (R^2^ = 0.883), with a corresponding *p*-value of 0.988 indicating that the results from each methodology are the same (Figure 1). We are therefore confident that a good representation of transcript levels in both environments was obtained using the presented transcriptome sequencing methodology.

**Table 1:** Summary of unfiltered transcriptome sequencing data.

	**Beer 1**	**Beer 2**	**MRS-B 1**	**MRS-B 2**
# reads ^a^	28,752,102	19,157,774	32,738,078	29,286,058
aligned	86.2%	85.9%	86.2%	85.7%
rRNA ^b^	64.1%	77.8%	35.7%	45.8%
reads in ORFs ^c^	95.5%	95.5%	94.8%	94.6%

^a^ Includes both reads from a pair.

^b^ Of all reads aligned, percentage that is to rRNA genes.

^c^ Of all non-rRNA and - tRNA reads, percentage that is found in annotated open reading frames.

### Metabolite transport and utilization

**Figure 2:**
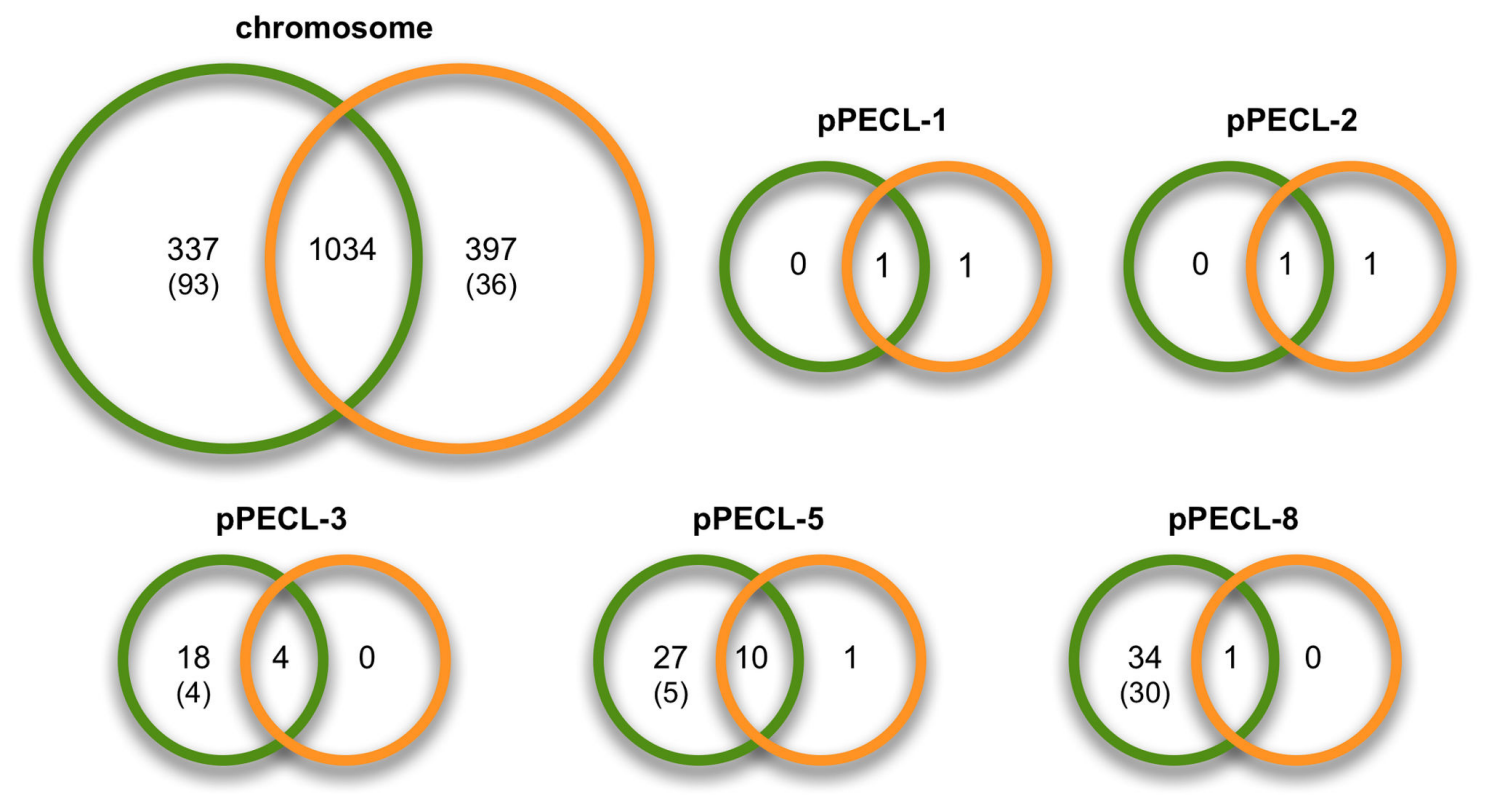
Summary of transcripts showing significantly different levels in beer or MRS-B. Each genetic element is depicted with two overlapping circles. A green circle represents the number of transcripts showing significantly higher levels during Pc344-358 growth in beer, whereas the orange circle shows the same thing in MRS-B. Numbers inside parentheses indicate how many significant differences were greater than 4-fold. The number inside the overlapping portion of the two circles gives the number of transcripts that did not show significantly different levels in the two environments, based on a *p*-value cutoff of 0.01.

**Figure 3:**
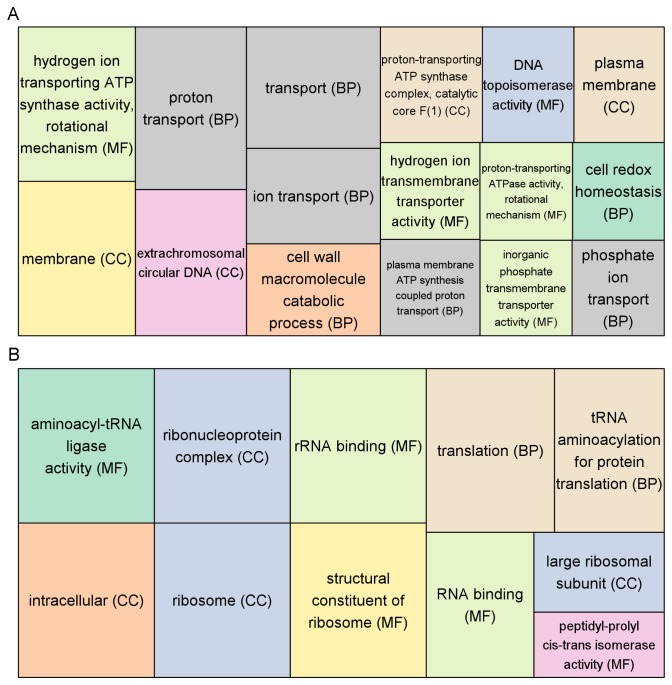
GO term analysis. Enriched GO terms were found in groups of genes showing significantly higher transcript levels in beer (A) and in MRS-B (B). Each GO term is provided with its corresponding ontology category (BP = biological process; CC = cellular component; MF = molecular function). Only GO terms showing over-representation with a *p*-value < 0.05 (determined by GOseq) are depicted, with the size of each rectangle reflecting the associated *p*-value. Similar GO terms are visualized in the same color.

Because this is the first description of a bacterial transcriptome during growth in beer, we compared gene expression levels to those found during growth in a nutrient-rich laboratory medium. Our goal was to establish a foundation upon which future, more targeted analyses can be based (e.g., investigating genes involved in hop resistance). We used a stringent *p*-value cut-off of 0.01 to determine statistically significant differences in transcript levels between the two growth environments. As shown in Figure 2, approximately one third of the chromosomal genes had significantly different transcript levels in one of the two environments, with 325 showing higher transcription during growth in beer. Of those 325 genes, 95 demonstrated at least 4-fold higher transcript levels in beer than in MRS-B. We applied the program GOseq [16] to find enriched gene ontology (GO) terms in groups of genes showing significantly higher expression in either growth medium. Taking gene length into account to prevent biases, GOseq indicated 27 GO terms that were enriched in the subsets of genes showing significantly different transcript levels in Pc344-358 growing in beer and in MRS-B (Figure 3).

**Figure 4:**
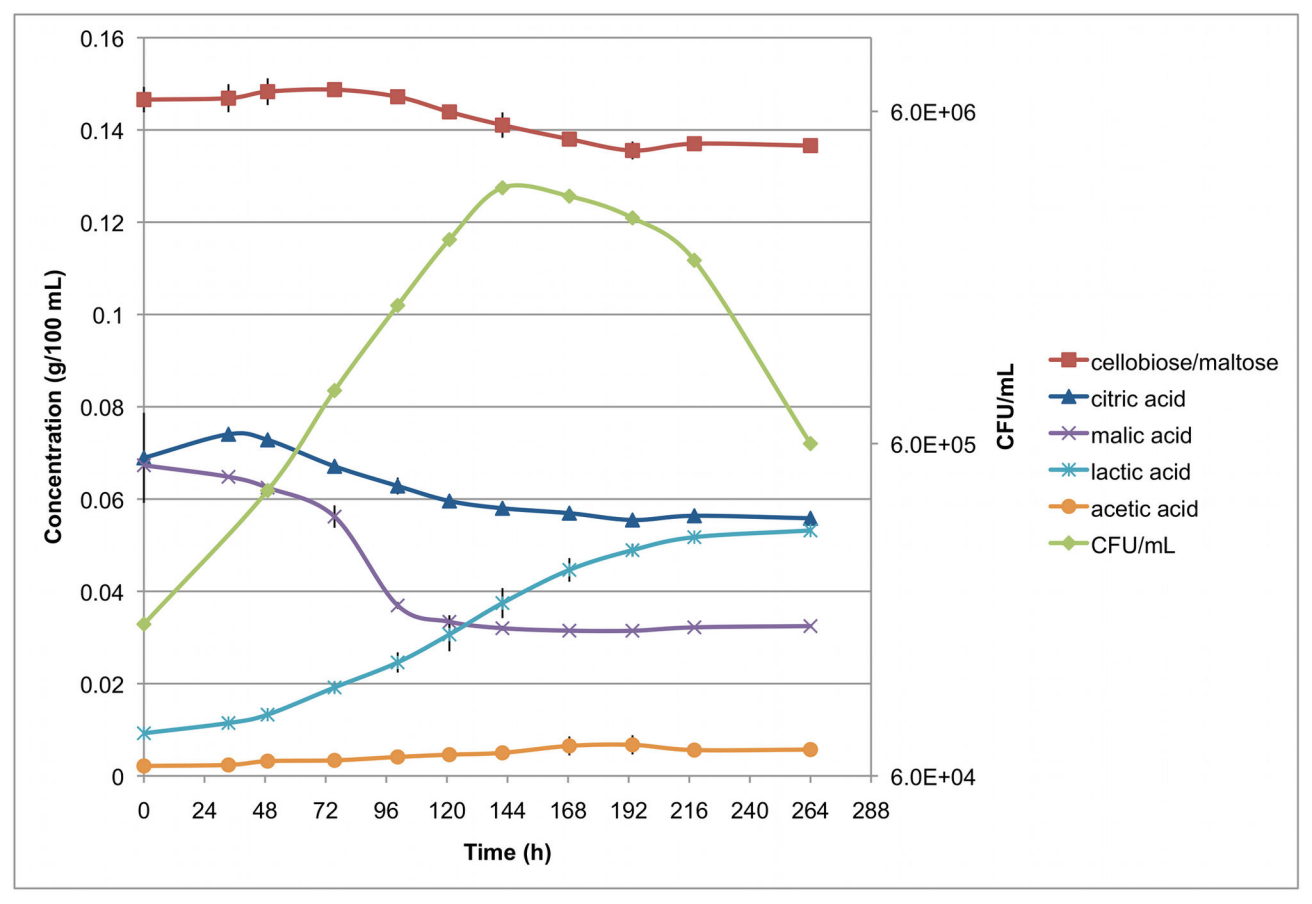
HPLC analysis of beer during Pc344-358 growth. Estimated concentrations of each compound were determined over time and plate counts were used to measure bacterial growth. Triplicate growth curves were analyzed, and standard deviations are indicated with error bars. Cellobiose and maltose could not be differentiated on the HPLC column, and are thus grouped together. Dextrin, ethanol, and glucose data are not included, as no change in concentration was found, or it was too low to detect (i.e., glucose).

The largest differences in transcript levels in either medium were found for genes listed in Table 2, showing that several operons are very important for Pc344-358 during growth in beer. The first two of note are the citric acid and malolactic fermentation operons (i.e., the *mleAP* and *citPCDEFXG* genes), which show 30- to 70-fold and ~20-fold higher transcript levels during mid-exponential growth in beer, respectively. To confirm these results, high-performance liquid chromatography (HPLC) analysis was performed on beer sampled daily during Pc344-358 growth (Figure 4). Levels of the organic acids malate and citrate were found to decrease during growth of Pc344-358, while lactic acid levels increased. In addition, the carbohydrates maltose and/or cellobiose were shown to decrease over time (the HPLC analysis could not differentiate these two disaccharides). A 38-fold increase in transcript levels was found for a cellobiose phosphotransferase system (PTS) transporter and a glucosidase that is presumably responsible for cellobiose breakdown. As no genes for maltose utilization showed increased expression, the carbohydrate measured by HPLC analysis was most likely cellobiose, not maltose.

**Table 2:** Summary of top twenty significantly different Pc344-358 transcript levels during growth in beer and in MRS-B.

**Locus_tag**	**Gene**	**Description**	**Fold Increase**	**Location**
**Higher transcript levels in beer**				
PECL_1707	*aguD*	agmatine/putrescine transporter	269	chromosome
PECL_1706	*aguA1*	agmatine deiminase	202	chromosome
PECL_1959		putative ncRNA	157	pPECL-8
PECL_1708	*aguB*	putrescine carbamoyltransferase	90	chromosome
PECL_1505	*mleP*	malate permease	69	chromosome
PECL_1705	*aguC*	carbamate kinase	64	chromosome
PECL_2060		putative ncRNA	64	chromosome
PECL_1438		cellulase family protein	37	chromosome
PECL_1437		PTS, cellobiose-specific IIC component	37	chromosome
PECL_2031		hypothetical protein	33	pPECL-8
PECL_2059		putative ncRNA	32	chromosome
PECL_1506	*mleA*	malolactic enzyme	27	chromosome
PECL_1605		prolyl oligopeptidase family protein	25	chromosome
PECL_256	*citF*	citrate lyase, alpha subunit	23	chromosome
PECL_255	*citE*	citrate (pro-3S)-lyase, beta subunit	22	chromosome
PECL_1516	*eno*	enolase (phosphopyruvate hydratase)	22	chromosome
PECL_257	*citX*	holo-ACP synthase	21	chromosome
PECL_258	*citG*	triphosphoribosyl-dephospho-CoA synthase	20	chromosome
PECL_253	*citC*	[citrate (pro-3S)-lyase] ligase	18	chromosome
PECL_1554		hypothetical protein	16	chromosome
**Higher transcript levels in MRS-B**				
PECL_341		PTS, mannose-specific IIC component	14	chromosome
PECL_340		PTS, mannose-specific IIAB component	11	chromosome
PECL_342		PTS, mannose-specific IID component	10	chromosome
PECL_737	*gla*	glycerol facilitator-aquaporin	10	chromosome
PECL_873		hypothetical protein, possibly prophage	8	chromosome
PECL_1161		cyclic nucleotide-binding domain protein	8	chromosome
PECL_1362		amino acid transporter	8	chromosome
PECL_1160	*argR*	arginine repressor	8	chromosome
PECL_1851	*pbuX*	xanthine permease family protein	8	chromosome
PECL_1742		putative membrane protein	7	chromosome
PECL_874		hypothetical protein, possibly prophage	7	chromosome
PECL_123		hypothetical protein	7	chromosome
PECL_1361		phosphoglycerate mutase	7	chromosome
PECL_56	*tyrS*	tyrosine--tRNA ligase	7	chromosome
PECL_875		prophage Lp1 protein 2	7	chromosome
PECL_1799		hypothetical protein	6	chromosome
PECL_1777	*nagE*	PTS, N-acetylglucosamine-specific IIABC component	6	chromosome
PECL_876		prophage Lp1 protein 6	6	chromosome
PECL_1175		DNA/RNA non-specific endonuclease family protein	6	chromosome
PECL_47		hypothetical protein	6	chromosome

Several other operons showing higher Pc344-358 transcript levels during growth in beer revolve around transport and utilization of various substrates (Table S3). Some encode the machinery for transport of phosphate (*pstSCAB*), D-methionine (PECL_1523-1526), and possibly glutamine (PECL_1446-1449), as well as proteins that are potentially involved in a thiamine transport and salvage pathway (PECL_1555-1561). Three operons were further investigated as potential nutrient acquisition mechanisms being used by Pc344-358 in the nutrient-limited beer environment. Specifically, genes involved in the uptake of mannitol (*mltAFD*), glycerol/dihydroxyacetone (*glpF/dhaKLM*), and trehalose (*treBC*) demonstrated higher transcript levels in beer, which led us to perform an HPLC analysis of beer sampled at two time points for these compounds (0 and 76 hr growth, the latter being the same time point used for RNA-sequencing). No mannitol or trehalose was detected (possibly present at too low concentrations for detection by HPLC) and no change in glycerol levels was found after growth of Pc344-358. It is therefore possible that these operons are very tightly repressed during growth in MRS-B, as very few sequencing reads represent these genes in the MRS-B samples. Alternatively, the higher operon transcript levels may be due to specificity for other substrates found in beer, especially considering both GlpF and DHA kinase have demonstrated specificity for other molecules [17,18].

Genes in the agmatine deiminase (AgDI) operon (*agu*) also demonstrated anywhere from 15- to 270-fold higher expression in beer when compared to MRS-B (Table 2). The AgDI pathway is involved in agmatine import, with subsequent deimination and decarboxylation, eventually leading to the production of ATP, CO_2_, putrescine, and ammonia. The uptake of agmatine is coupled with putrescine export, providing an energetically favorable way to create ATP (i.e., none of the components created by the pathway are used for biosynthesis, other than the produced ATP) [19]. Not only is the AgDI pathway a means for energy production, but it also helps regulate pH (especially in the presence of the ionophore hops) through the production of ammonia. A very similar pathway involving arginine utilization and ornithine production (the arginine deiminase (ADI) pathway) has previously been implicated in hop resistance in a *Lactobacillus* *brevis* isolate [20]; however, the ADI pathway is not found in Pc344. As such, it appears as though Pc344 is using other compounds in beer to produce energy and counteract the effect of hops. Here it should be noted that varying amounts of agmatine are present in a range of beer styles worldwide [21–23].

### Transcriptional response to hops

Hop compounds can inhibit gram-positive bacterial growth by interfering with cell wall permeability [24,25], via their ionophore activity [26], and/or by creating oxidative stress through manganese-complexes [27,28]. Most gram-positive bacteria are unable to grow in the presence of hops unless mechanisms are in place to overcome the aforementioned stresses. Modes of hop resistance may therefore be classified into: 1) increased rate of proton expulsion [29], 2) ATP-dependent multidrug resistance transporters such as *horA* [10], 3) cell membrane modification [20], and 4) the oxidative stress response [27,28]. Considering few of these functions would be required for growth in MRS-B, the GO terms highlighted in genes preferentially expressed while Pc344-358 is growing in beer are not surprising (Figure 3a).

To overcome the ionophore activity of hops, several proton-motive force- (PMF) regulating genes show higher expression in beer, including the proton-transporting ATPase machinery (*atp* operon). The previously discussed decarboxylation antiporter systems and malolactic and citric acid fermentation operons are also presumably involved in counteracting hops (and low pH) via the buffering capacity and PMF generated by these pathways. Another mode of resistance that has been demonstrated in LAB is exporting hop-compounds from the cell. Two of the known BSR genes *horA* and *horC* are ATP-binding-cassette (ABC) and PMF-dependent multidrug transporters, respectively, that are proposed to export hops from the cell [10,12]. Pc344-358 only contains one known BSR gene, *horA*. Our transcriptome results provide further evidence for the potential role of *horA* in hops transport with the 11-fold higher transcript levels in Pc344-358 growing in beer than in MRS-B (Table S4). Additionally, one other ABC multidrug transporter (PECL_1630) demonstrates 8-fold more transcripts in beer. Further investigation of this gene is needed to elucidate if it also plays a role in enabling BSOs to grow in the presence of hops by exporting hop-compounds from the cell.

Although elongation of fatty acids (or membrane modification in general) has been shown to be a bacterial mechanism for overcoming the effects of hops [20] and ethanol (reviewed by [30]), modification of membranes by BSOs during growth in beer has never been analyzed. The transcriptome sequencing results presented here provide the first indication of membrane modification as a response to the beer environment, where lower levels of hops and ethanol (in comparison to levels analyzed in [20] and [30]) are encountered in combination as stresses. Evidence for this contention is the 2- to 3-fold higher expression of the fatty acid biosynthesis *fab* operon in Pc344-358 growing in beer (Table S3), despite much slower growth in this medium. It is also noted that the signal peptidase II gene responsible for lipoprotein export showed 1.7-fold higher expression in beer than in MRS-B, whereas the signal peptidase I gene responsible for secreted or membrane-bound protein export showed the opposite effect (decreased by 50%). Of further note is the expression level of genes surrounding the hop-resistance gene *horA* (present on pPECL-8 in Pc344-358). It has been noted previously that five genes are consistently found in the same order around the *horA* gene in BSOs, whereas the remainder of the plasmid carrying *horA* is rarely conserved [7,31–33]. Four of the five genes surrounding *horA* are involved in phospholipid or cell wall biosynthesis. Each has much higher (>3-fold) transcription in beer than *horA*, demonstrating some of the highest transcript levels of all genes on plasmid pPECL-8 (Table S4). This clearly indicates that further investigation into the role of these genes is warranted, not only to provide additional insight into how BSOs adapt to the stresses found in beer, but also to explore the potential expansion of current BSO detection techniques (i.e., if these genes are found in LAB BSO isolates that do not have *horA* or other known BSR genes).

Finally, a number of genes show higher transcription during growth of Pc344-358 in beer as a response to the oxidative stress imposed by hops. These include manganese transport proteins (PECL_313 and PECL_638), the methionine sulfoxide reductases MsrA and MsrB, as well as other metal transport and homeostasis proteins (PECL_793, PECL_1579, and PECL_1580). Pc344-358 also contains two genes potentially encoding a glutathione reductase (PECL_1643 which is chromosomal, and PECL_1987 which is on pPECL-3) that demonstrate 9- to 10-fold higher expression in beer. Also on plasmid pPECL-3 is a putative DNA protection during starvation (dps) protein that has very high expression in beer (13-fold higher than in MRS-B), and may be important for DNA protection during oxidative stress [34]. Lastly, genes encoding the nucleotide excision repair system (*uvrAB*) demonstrated significantly higher transcript levels in beer, presumably as a general response to DNA damage. Ultimately, the transcriptome sequencing results of Pc344-358 support that a multi-factorial response is needed to overcome the various stresses imposed by hops.

### Pc344-358 transcriptome during growth in MRS-B

The complementary side of our transcriptome sequencing experiment was to analyze gene expression during growth in a nutrient-rich laboratory medium. Enriched GO terms for the MRS-B environment were found to mostly involve translation (e.g., ribosomal constituents, rRNA binding, tRNA aminoacylation; Figure 3b). Comparing this to transcript levels found for individual genes, it is noted that most ribosomal protein genes have approximately half the expression level in beer of what is found during growth in MRS-B (Table S3). Since Pc344-358 grows more rapidly in MRS-B than in beer, this agrees with what occurs in *Escherichia coli* where ribosome synthesis is coupled to growth rates (i.e., faster growing cells produce more ribosomes) [35].

Not surprisingly, genes involved in glycolysis and uptake of certain carbohydrates (e.g., N-acetylglucosamine, mannose, sorbose), and in pyrimidine biosynthesis demonstrate higher expression during growth of Pc344-358 in MRS-B (Table 2). As well, genes associated with osmoprotection (e.g., osmosensory transporters) are preferentially transcribed during growth in MRS-B (Table 2). It is also interesting that several prophage genes have significantly higher transcript levels in MRS-B (Table 2), possibly indicating that their transcription is repressed in the stressful beer environment. Finally, it is worth noting that a lot of the highly transcribed genes in MRS-B encode hypothetical proteins with unknown function (Table S3), many of which are conserved in other LAB and therefore may have a basal role during growth of these organisms in nutrient-rich environments.

### Plasmid-based response to growth environment

It was hypothesized and then demonstrated that close proximity of cells in biofilms leads to increased plasmid transfer (e.g., [36] and reviewed in 37). Since the natural environment of Pc344 in a brewery most likely involves complex communities in biofilms, we were particularly interested in the role of mobile genetic elements during growth in beer. It was initially presumed that seven plasmids were present in the isolate used for RNA extraction and transcriptome sequencing when identical growth curves to that for Pc344NR were obtained in both MRS-B and beer. However, it was noted after aligning reads that plasmids pPECL-4 and pPECL-6 showed essentially no coverage. Genes on pPECL-4 encode several plasmid-associated and hypothetical proteins, as well as a putative glycosyl hydrolase, multicopper oxidase, and major facilitator superfamily permease, some of which could be beneficial for growth in beer. Similarly, pPECL-6 encodes a putative lantibiotic synthesis and transport system, and eight conserved hypothetical proteins with unknown function. We were therefore interested in whether or not these plasmids had been lost by Pc344NR during growth in preparation for sequencing, or if essentially no gene transcription from these two plasmids occurred during growth in either environment. Further investigation concluded that the two plasmids had indeed been lost prior to sequencing (data not shown), which together with the growth in beer data pointed to plasmids pPECL-4 and pPECL-6 having minimal roles in Pc344’s ability to grow in beer. To test this, quantitative PCR (qPCR) analysis was performed on cDNA obtained from Pc344NR growing exponentially in beer and in MRS-B. No significant differential gene expression was found for two and three genes on pPECL-6 and pPECL-4, respectively (data not shown; see Table S1 for targeted genes). This is in stark contrast to the transcriptome sequencing results for three of the five remaining plasmids in Pc344-358, where the majority of their genes show significantly higher transcript levels in beer (Figure 2). Conversely, the two small cryptic plasmids had minimal transcript level changes in either medium, which is not surprising considering each genetic element only contains two genes responsible for plasmid replication and copy number control.

Because Pc344 appears to rapidly change plasmid composition, we were curious if altered copy numbers of the five plasmids in Pc344-358 were responsible for the plasmid transcript level differences described here. We therefore analyzed the plasmid copy number (PCN) of each genetic element in Pc344-358 at mid-exponential growth in beer and MRS-B. qPCR was used to determine the absolute and relative quantity of each plasmid, with PCN calculated according to the number of detected chromosomes (using two single copy chromosome-localized genes *bsrA* and *gmk*). Similar to what was found with transcriptome sequencing results, the two small cryptic plasmids showed only minimal changes in copy number during exponential growth in MRS-B and beer (Table 3). In contrast, pPECL-3, pPECL-5, and pPECL-8 had slightly larger changes, with each being found at approximately double the copy number in Pc344-358 growing in beer. Considering the energy limitations imposed by the lack of nutrients in beer and the energy needed to overcome the antimicrobial effects of hops, it is interesting to find these plasmids are maintained at a slightly higher copy number in this environment. This finding indicates that the increase in transcript levels of genes found on pPECL-3, pPECL-5, and pPECL-8 cannot be solely attributed to transcriptional changes. Nonetheless, the overall increase in plasmid transcripts and copy number emphasizes the importance of these plasmids for Pc344-358 growing in beer.

**Table 3:** PCN for *P* *. claussenii* growing in beer and MRS-B.

**Target**	**PCN in beer ^a^**	**PCN in MRS-B ^a^**	**Fold Change ^b^**
pPECL-1	5.5 ± 0.6	4.0 ± 0.7	1.4 ± 0.3
pPECL-2	5.3 ± 0.8	7.7 ± 1.1	0.7 ± 0.1
pPECL-3	3.9 ± 0.7	1.6 ± 0.2	2.4 ± 0.6
pPECL-5	1.4 ± 0.2	0.7 ± 0.1	1.9 ± 0.5
pPECL-8	0.9 ± 0.1	0.4 ± 0.1	2.1 ± 0.5

^a^ PCN ± standard deviation; based on absolute quantification of plasmid-localized gene compared to chromosome-localized genes *bsrA* and *gmk* (i.e., number of plasmid copies per detected chromosome)*.*

^b^ Fold change in PCN ± standard deviation (beer compared to MRS-B); based on absolute quantification method (relative quantification results are not provided as they demonstrated the same fold changes).

For plasmids, the final question then becomes what transcript level changes are of prime importance for Pc344-358 growing in beer? As Table S4 illustrates, an assortment of genes are found on pPECL-3, pPECL-5, and pPECL-8, with most showing higher transcript levels in beer than in MRS-B. Plasmid pPECL-5 appears to contain genes relevant to conjugation, and may therefore not only have a role in Pc344-358’s ability to grow in beer, but possibly also in enabling genetic transfer among LAB and thus emergence of new BSOs. pPECL-8 not only has the previously mentioned BSR gene *horA* and surrounding membrane modification genes, but it also contains 30 genes that show at minimum ~4-times higher transcript levels in beer than what is seen in MRS-B. Similarly, the largest transcript level differences for genes on pPECL-3 correspond to an 8- to 13-fold increase of a hypothetical protein (PECL_1981), and the previously mentioned oxidative stress response glutathione reductase and DNA protection during starvation proteins. Lastly, it should be noted that all three plasmids code for a large number of hypothetical proteins, few of which have homologs in other LAB.

### Putative non-coding RNAs

At a genome-wide level, it is noteworthy that some of the most highly transcribed genes during growth in beer are potentially non-coding RNAs (ncRNA) and hypothetical proteins (Tables S3, S4, S5). Due to the hypothetical nature of these small proteins and ncRNA, however, their role in Pc344-358 while growing in beer remains unknown. One possibility for ncRNA is involvement in plasmid replication (reviewed in 38), which may be why Pc344-358 plasmid-expressed ncRNA have such high levels in beer. Alternatively, beer-induced hypothetical proteins and ncRNA may have a role in regulating gene expression in response to stresses, which is an area in general that needs further investigation in LAB.

When transcript levels for all Pc344-358 genes are compared, it is noted that four of the most highly expressed genes in beer are ncRNA (Table S5). Ribonuclease P (PECL_2058), which is involved in tRNA maturation, showed 3-fold higher levels when Pc344-358 is growing in beer, while 11-fold higher levels were found for the transfer messenger RNA (tmRNA) *ssrA* gene (PECL_2056). The latter bifunctional tmRNA is involved in trans-translation and some gene regulation (reviewed by [39]). The process of trans-translation rescues stalled ribosomes from damaged mRNA and requires a 1:1:1 ratio of tmRNA complexed with the SmpB and elongation factor Tu (EF-Tu) proteins [40]. However, unlike tmRNA, the *smpB* gene (PECL_556) actually shows 2.6 times higher transcript levels in Pc344-358 growing in MRS-B, while the EF-Tu gene (PECL_851) shows no significant difference in either medium. As such, it is unclear if trans-translation is indeed occurring at a higher frequency when Pc344-358 is growing in beer, or if an additional role for tmRNA (or an alternative mode of tmRNA regulation) exists in this stressful environment.

Two other putative ncRNA genes also have very high transcript levels in beer (PECL_2059 and PECL_2060), yet show no similarity to previously described genes or to any RNA families in the Rfam database. These two structures show 32- and 64-fold higher levels when Pc344-358 is growing in beer (Table S5), indicating that their role is important for this bacterium in this environment. Because of the uncertainty with library preparation biases for small RNA molecules, we analyzed the expression of PECL_2060 (188 bp) as one of the nine genes confirmed with RT-qPCR. The independent analysis demonstrated 12 ± 7 fold higher expression of PECL_2060 in beer (*p-value* 0.025). This is a smaller difference than what was obtained with the transcriptome sequencing results; however, it is also noted that the transcriptome sequencing beer biological replicates showed a large variability in the number of reads for PECL_2060 (RPKM values of 1.9x10^5^ and 7.2x10^4^ for beer biological replicates, versus 3.2x10^3^ and 2.0x10^3^ for MRS-B). As such, the transcriptome sequencing results for PECL_2060 may be artificially high based on the discrepant beer biological replicates, possibly as a result of library preparation biases, or high turnover of this transcript. In any case, the RT-qPCR analysis confirms a significant and substantial increase of PECL_2060 during growth in beer. Predicting putative regulatory targets for these ncRNA has not been possible as the Pc344 genome was only recently released and is not yet represented in programs performing target prediction of small RNAs. Nevertheless, we have shown that several ncRNA and hypothetical proteins have high expression when Pc344-358 is growing in beer, putting these genes at very high interest for follow-up analyses involving targeted questions (e.g., role in hop resistance) in a range of BSOs. Investigation of these genes can also be broadened to other LAB to help elucidate potential roles in the global stress response of these organisms.

## Conclusions

Because *P* *. claussenii* is a difficult organism to work with from the perspective of genetic manipulation (e.g., knockout or cloning based studies), we used transcriptome sequencing to determine which genes are used during growth in the harsh environment of beer as compared to growth in the nutrient-rich MRS-B environment. The results provide the first description of *P* *. claussenii* gene expression in response to various stresses and low nutrients. Overall, our results support the concept that a multi-factorial response is needed to cope with the numerous conditions in beer that can negatively impact growth. How the general stress response relates to the mechanisms allowing *P* *. claussenii* to grow in beer is still unclear. Nonetheless, the RNA-seq results presented here strongly indicate several new targets for investigation in *P* *. claussenii* (and LAB generally) when grown not only in beer, but also in other stress conditions. The identified genes are predicted to be involved in cell membrane modification and, in addition, specify a variety of hypothetical proteins and putative ncRNA.

## Materials and Methods

### Isolates

The original *P* *. claussenii* (Pc344) isolate was obtained as a beer-spoilage isolate [8] and is available in several culture collections (ATCC BAA-344^T^, DSM 14800^T^, VTT E-032355^T^). The non-ropy variant Pc344NR was obtained by repeated subculturing in MRS-B as described in [14], which resulted in the loss of the *gtf* gene-containing plasmid pPECL-7. The isolate used for transcriptome sequencing was initially thought to contain seven plasmids (i.e., Pc344NR, containing pPECL-1 through pPECL-6, and pPECL-8). However, it was determined after transcriptome sequencing had been performed that the isolate had lost two plasmids during initial propagation in MRS-B leading into the preparation of both beer and MRS-B transcriptome sequencing samples. As such, transcriptome data was obtained on the plasmid variant Pc344-358, which is lacking plasmids pPECL-4, pPECL-6, and pPECL-7.

### Growth conditions

Pc344-358 was grown in beer and MRS-B [41] to analyze transcript and plasmid levels in both environments. A lager beer with 5% (v/v) alcohol, pH 4.2, and roughly 11 hop-bitterness units was used, while the MRS-B was kept at pH 6.5. Growth curves were first established in each medium to determine the mid-exponential stage of growth. In MRS-B, an overnight culture was inoculated at 1% (v/v) into fresh medium, and growth at 30°C was monitored every hour for the first 12 hr, followed by every four hours for an additional 16 hr. At each time point, the optical density (A_600nm_) and colony-forming units (CFUs) were determined. Mid-exponential growth was found to correspond with an OD of 0.47 (~1x10^8^ CFU/mL). A similar growth curve analysis was done in beer; however, Pc344-358 had to first be acclimatized to grow in this harsh environment [9]. As such, 25 µL of an overnight culture in MRS-B was transferred into 85% beer + 15% 2x modified MRS-B (no Tween) medium (12 mL) and grown for 1 day. This was then used to inoculate 100 µL into 100% beer (3 x 12 mL tubes), followed by a 5-day incubation at 30°C. The final inoculation was done into 1 L of beer (a 3.3% inoculum of the 5-day culture) and growth was monitored every 24 hr by plating on MRS agar for 11 days. Three biological replicates were performed, and samples were also taken daily for HPLC analysis. Mid-exponential phase growth was found to correspond to 76-83 hr after inoculation (~1x10^6^ CFU/mL). It is therefore emphasized that the same phase of growth (e.g., mid-exponential) corresponds to different growth rates and cell concentrations in MRS-B and beer, with Pc344-358 dividing at a much faster rate in the former medium.

For transcriptome sequencing and PCN experiments, two or three biological replicates, respectively, were created with cells at mid-exponential growth in each medium. Negative controls were also included for each medium (i.e., no organism was added), and neither demonstrated visible growth or any CFU formation on MRS-B agar plates throughout the RNA-seq experiment. Once the exponential growth phase was reached, cells in MRS-B were harvested by centrifugation at 10,000 *x g* for 3 min. For beer samples, four 250 mL aliquots were centrifuged for 10 min at 4,000 *x g*. Each beer sample pellet was then resuspended in approximately 10 mL of the growth medium and pooled, with the cells finally collected by centrifugation at 10,000 *x g* for 3 min. All cell pellets were flash-frozen in liquid nitrogen and stored at -80°C until RNA or DNA extractions were performed.

### HPLC analysis

During the growth curve analysis of Pc344-358 in beer, the medium was analyzed daily for carbohydrate, organic acid, and ethanol content. Samples were filtered with a 0.45 µM syringe filter, and then analyzed on a Waters HPLC System (RI detection, with Breeze software) using a Phenomenex Rezex RHM-monosaccharide H^+^ (8%; 300 x 7.80 mm) column with a 0.5 mM H_2_SO_4_ mobile phase (flow rate 0.5 mL/min). Ten µL of sample was separated in a 30 min run at 65°C, and concentrations of the following compounds were determined: acetic acid, cellobiose, citric acid, dextrin, ethanol, glucose, glycerol, lactic acid, malic acid, maltose, mannitol, and trehalose. All standards were prepared in HPLC-grade water. Maltose and cellobiose were grouped together in the analyses, as complete separation of these two compounds was not achieved.

### RNA isolation

A combined TRIzol- and column-based method was used to extract RNA from Pc344-358. MO BIO UltraClean Microbial RNA kit columns were chosen as they purify total RNA (i.e., there is no size exclusion). Pellets were thawed in 1 mL of TRIzol (Invitrogen) and subjected to five-1 min bead-beating cycles using a vortex, which were each followed by 1 min on ice (0.1 mm glass beads, BioSpec Products). The RNA in the aqueous phase after phase separation was then purified using the UltraClean Microbial RNA Isolation Kit (MO BIO). A 15-min on-column DNase digestion was included in the protocol (On-Spin Column DNase I Kit, MO BIO). Following RNA isolation, a second DNase digestion was performed to remove any remaining DNA (12 U TURBO DNase, Ambion) and a MO BIO RNA Kit column was used for post-DNase clean up. RNA quality was assessed with UV spectrophotometry, Bioanalyzer (Agilent) analysis, and gel electrophoresis.

### mRNA purification and sequencing

To verify complete DNA removal in the RNA samples, cDNA and no-reverse transcription (noRT) controls were evaluated by qPCR. cDNA was prepared using the SuperScript III First-Strand Synthesis SuperMix for qRT-PCR kit (Invitrogen), while reverse transcriptase was replaced by water in the noRT controls. qPCR was performed in a MasterCycler RealPlex^4^ (Eppendorf) using the FastStart Universal SYBR Green Master (Rox) kit (Roche). Primers for the 16S rRNA gene (386F/534R [42,43]), and the *recA* and *horA* genes (Table S1) were used. No amplification in the noRT controls indicated that all residual DNA had been removed. After verification, total RNA was enriched for mRNA via the MICROB*Express* Bacterial mRNA Enrichment Kit (Ambion) using the manufacturer’s instructions. Roughly 1 µg and 2-5 µg of RNA were used for beer and MRS-B samples, respectively. The mRNA-enriched samples were run on a Bioanalyzer (Agilent) to ensure that most of the rRNA had been removed. RNA sequencing was done using the Illumina TruSeq RNA Sample Preparation Kit and GAIIx technology at the Genomic Sciences Laboratory, North Carolina State University in Raleigh, NC. All library fragment sizes were ~140-290 bp, which provided final library distributions of 335 bp ± 75 bp (including 2 x 60 bp adapters). All four cDNA samples (two biological replicates of cells grown in each medium) were multiplexed in one lane and 68 bp paired-end reads were obtained. All raw data is available at the NCBI Sequence Read Archive under the BioProject number PRJNA185806, with sequences for each biological replicate found under the accession numbers SRX216314 and SRX216316 for the beer samples, and SRX216317, SRX216319 for the MRS-B samples.

### Transcriptome sequencing

Because a good quality genome sequence for Pc344 was available, we investigated the effect of quality filtering reads on transcript level results. Stringent parameters were used for quality filtering to provide high quality libraries for comparison to unfiltered reads. The FASTX-Toolkit (http://hannonlab.cshl.edu/fastx_toolkit/) was used to perform the following filtering steps: Illumina adapter sequences were first removed and reads were then trimmed from each end until a base with Phred quality score > 30 was reached. Any reads less than twenty nucleotides after trimming were discarded and remaining reads were kept only if > 90% of the read had a Phred quality score > 20. Any reads not meeting these criteria were discarded.

Reads were aligned to the *P* *. claussenii* ATCC BAA-344^T^ genome ( [7], BioProject Accession No. PRJNA81103), using Bow tie 2 (version 2.0.0-beta 5) [44]. For unfiltered libraries, each paired-end read was provided to Bow tie 2 to run in -M mode (search for multiple alignments, report the best one) with end-to-end alignments using the -- very-sensitive parameter and a maximum fragment length set to 400 (-X option). The same settings were used for the quality-filtered version of the library, however, reads that no longer had a “mate” (because it was discarded) were provided as unpaired reads. Following alignment, each annotated gene was provided as input to the Bioconductor (release 2.10) R program GenomicRanges, which summarizes the number of reads aligned to each feature via the summarizeOverlaps function in “Union” mode [45]. Differential expression analysis was then performed on these raw read counts via *DESeq* (version 1.6.1) [46]. Genes showing an adjusted *p*-value (for multiple testing via the Benjamini-Hochberg procedure) less than 0.01 were considered to have significantly different transcript levels in the two growth environments and were therefore included in further investigations. As well, 8 genes with adjusted *p*-values < 0.05 were included as they demonstrated strong evidence for differential expression (i.e., were part of an operon). Groups of genes showing significantly different transcript levels in either growth environment were assessed for enriched GO terms via the Bioconductor R package GOseq (version 1.6.1) [16]. GOseq results were then visualized with the TreeMap program in REVIGO [47], with settings for large similarities allowed and category sizing based on GOseq provided *p*-values.

In addition to transcript level analyses, the transcriptome sequencing data was used to finish assembling the eighth plasmid (pPECL-8) in the *P* *. claussenii* genome sequence (originally only ~20 kb were available [7]). The *de novo* transcriptome assembly pipeline Rnnotator was used to build contigs from the RNA-seq reads [48]. Contigs not already present in the Pc344 genome sequence were proposed to be part of pPECL-8, except for several contigs representing rRNA from *Saccharomyces* that were assembled from the beer samples. PCR-based gap closing was performed to join contigs, and sequencing was done to verify and complete the final plasmid (33,246 bp; updated in GenBank, Accession No. CP003145.2).

### Annotation verification

Output from Bow tie 2 (SAM format) was converted to sorted, indexed BAM files via the SAMtools package [49], and reads aligning to the Pc344 genome were then visualized with Artemis [50]. To identify missing genes, the genome was scanned for regions showing read coverage, but no corresponding annotation. Potential ORFs were then used to BLAST the NCBI database to determine if the expressed region corresponded to a coding sequence. Sequences were also used to search the Rfam database [51] for potential non-coding RNAs.

### RT-qPCR comparison to RNA-seq

Transcriptome sequencing results were verified via RT-qPCR analysis on RNA extracted at a later date. It was noted that the isolate used for RNA-sequencing was missing plasmids pPECL-4 and pPECL-6. We therefore ensured that the isolate used for RT-qPCR analysis had seven plasmids (i.e., Pc344NR, which is only missing the 7^th^ plasmid that confers the ropy-phenotype) by PCR detection of each plasmid. RNA was extracted via the methodology described above, however, the TURBO DNA-*free* kit (Ambion) was used instead of TURBO DNase, which eliminated the need for post-DNase cleanup via MO BIO RNA Isolation Kit columns. Three biological replicates of cells grown to exponential phase in each medium were used for RNA extraction. The cDNA was synthesized using 1 µg of RNA, Promega GoScript Reverse Transcriptase, and random hexamer primers as previously described [15].

Nine genes representing a range of RNA-sequencing results (i.e., significantly increased expression in beer and in MRS-B, as well as no difference) were analyzed by qPCR. Data for eleven additional genes from a previous study were also included, as all twenty genes were analyzed concurrently in the same cDNA samples [15]. We also assayed three and two genes present on plasmids pPECL-4 and pPECL-6, respectively, to assess if some of the genes of interest on these plasmids were differentially expressed during growth in beer. All primer set sequences are provided in Table S1, along with their respective PCR amplification efficiencies. For qPCR gene expression data normalization, we used the two previously determined reference genes *ldhA* and *gyrA* [15]. qPCR analysis (e.g., efficiency determination, differential gene expression calculation) was performed as previously described [15]. Briefly, the qPCR program consisted of 95^°^C for 30 sec; 40 cycles of 95^°^C for 5 sec, 55^°^C for 10 sec (with fluorescence data collection), followed by melt curve analysis (65-95^°^C with increments of 0.5^°^C/5 sec). Reactions were run in a MiniOpticon Real-Time PCR system (Bio-Rad) with the CFX Manager software (version 2.1) being used in regression mode to determine quantification cycles. Triplicate reactions were prepared for each sample using the SsoFast EvaGreen Supermix (Bio-Rad) with a final reaction volume of 15 µL, containing 2 µL template (~6 ng cDNA) and 0.3 µM primers.

### PCN analysis

The copy number of each plasmid in Pc344-358 was determined via qPCR in triplicate for mid-exponentially growing cultures in beer and MRS-B. DNA was extracted from pellets stored at -80°C using the MO BIO UltraClean Microbial DNA Isolation Kit as per the manufacturer’s protocol with the following modifications: the optional incubation at 70°C for 10 min was included for difficult-to-lyse cells, followed by a 5 min bead beating step on a flat-bed vortex to reduce shearing. To ensure proper quantification of plasmids when using a linear standard [52], DNA was digested with an enzyme known to cut each plasmid at least once. Three hundred ng of each DNA extraction was digested for 16 hr with 20 U MbiI (New England Biolabs) at 37°C, followed by heat inactivation at 65°C for 20 min. Because plasmid concentrations are not high enough for visualization on a gel, full digestion was ensured using PCR. Ten fold serial dilutions were prepared from digested and undigested DNA, and PCR was performed with primers spanning MbiI cut sites. Undigested plasmids were found to be ~1 in 1000, and thus considered to have a negligible impact on qPCR results.

qPCR was performed on six DNA extractions (three for each medium) in triplicate using the Bio-Rad MiniOpticon Real-Time PCR system as described earlier. Each reaction contained 6 ng of digested DNA, and each qPCR run included two inter-run calibrators for data normalization across runs (Table S1). PCN was assessed using relative and absolute quantification methods, with the two chromosome-localized genes *bsrA* and *gmk* (both single copy genes) being used as references. For absolute quantification, a standard curve of PCR amplicons from each plasmid was used. In short, amplicons produced using primers specific for each plasmid (Table S1) were purified and the concentration (pmol/μL) was determined via the GE NanoVue Spectrophotometer Oligo program which takes the oligo base-composition and length into account. The copy number of each amplicon was then determined and used to create five 10-fold serial dilutions ranging from 10^4^-10^8^ copies per qPCR. The standard curve was used to determine absolute PCN, as well as the PCR amplification efficiency (which was used for relative quantification). PCN was calculated by dividing the determined absolute PCN by the average absolute copy number of two chromosome-localized genes. For relative quantification, inter-run calibration and relative quantity calculations were done as described in [53], using both chromosome-localized genes as references.

## Supporting Information

Figure S1 **Comparison of biological replicate transcriptome sequencing results.** Log_10_ transformed RPKM values are plotted for each gene in each replicate for Pc344-358 grown in beer (A) and MRS-B (B).
(TIF)

Table S1 **qPCR primers used in this study.**
(PDF)

Table S2 **Genes added to the *P* *. claussenii* ATCC BAA-344^T^ genome annotation.**
(PDF)

Table S3 **Transcriptome-sequencing results from *DESeq* for chromosomal genes.**
(XLSX)

Table S4 **Transcriptome-sequencing results from *DESeq* for plasmid-localized genes.**
(XLSX)

Table S5 **All transcriptome-sequencing results from *DESeq*, ordered from high to low expression level in beer.**
(XLSX)
