# Supplementary figures and images for: Transcriptome Sequence and Plasmid Copy Number Analysis of the Brewery Isolate Pediococcus claussenii ATCC BAA-344T during Growth in Beer
Source: PLoS One. 2013 Sep 6;8(9):e73627. doi: 10.1371/journal.pone.0073627 (PMC3765258; doi:10.1371/journal.pone.0073627)

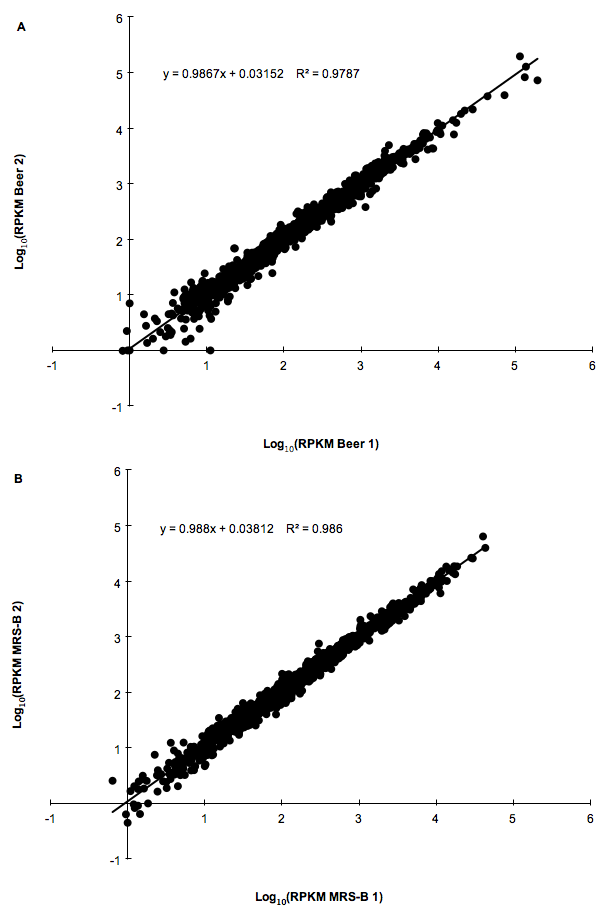

Supplement: Figure S1 — Comparison of biological replicate transcriptome sequencing results. Log10 transformed RPKM values are plotted for each gene in each replicate for Pc344-358 grown in beer (A) and MRS-B (B). (TIF) [file pone.0073627.s001.tif]
