# Supplementary material for: Transcriptome Sequence and Plasmid Copy Number Analysis of the Brewery Isolate Pediococcus claussenii ATCC BAA-344T during Growth in Beer
Source: PLoS One. 2013 Sep 6;8(9):e73627. doi: 10.1371/journal.pone.0073627 (PMC3765258; doi:10.1371/journal.pone.0073627)
Supplement: Table S1 — qPCR primers used in this study. (PDF) [file pone.0073627.s002.pdf]

Table S1. qPCR primers

Gene [a]	Fwd-primer	Rev-primer	Amplicon size (bp)	Efficiency [b]	Location	Gene length (bp)
Putative ncRNA						
PECL_1959	AGTAGCCTAAGTGTCCGTGTC	GACGGTGGCAGATTCTAACG	172	100 ± 4	pPECL-8	260
Malate permease						
mleP	ATGATGGCATTGGGATTTGG	ATGCTGGTAACGCAACATTCATG	99	102 ± 6	chromosome	968
Putative ncRNA						
PECL_2060	GGTCATTATCTAACCCAAGTAAGC	TCCATATTCTTCCGACAACATC	125	101 ± 2	chromosome	188
Malolactic enzyme						
mleA	CCACCTTTCGTTCAAACTTTAGATG	ACAATGTGTTGGCTAAACAGC	151	100 ± 2	chromosome	1616
Agmatine deiminase, copy 2						
aguA2	TGACGGGCTGTACTTTCCAT	CCTCACCATCAACCATCACT	108	106 ± 4	chromosome	1103
Cation transporting ATPase						
PECL_321	CACAAGTCTCATGGCTATTCTAC	GCATGTTCTTGCCAGAAGC	131	99 ± 3	chromosome	2792
Phosphomevalonate kinase						
pmk	GTGACAGCCAGCATCGAACC	TGCCGCTGAGCTACCCAAG	269	99 ± 7	chromosome	1082
Glycerol facilitator aquaporin						
gla	GCTATGGGTGTGGTGTAATG	GCCAGACAACTGTGCAAGAA	147	107 ± 3	chromosome	731
PTS, mannose-specific, IIAB component						
PECL_340	ACCTGACGACATTCACGCA	AGTTCAAGCCAGCAACGA	164	97 ± 4	chromosome	977
DNA gyrase, A subunit (reference gene)						
gyrA	GGTACGAATGGCACAGGAC	ACTGGTTCACGTTCTGAGC	204	99 ± 5	chromosome	2495
D-lactate dehydrogenase (reference gene)						
ldhA [c]	CTGGATTCTGAGACGCTGG	CATTAGGTGAATATGCTGGGAC	211	100 ± 4	chromosome	995
Glycosyl hydrolase, family 53						
PECL_1919	TCGCTACAAGATGCTGATGG	AGTAGAAGGCTCCAAGTCCA	131	114 ± 4	pPECL-4	5174
Putative multicopper oxidase						
PECL_1921	TATCGCCGTGGTCAACATAC	GCATCCGCCATCAACATAAG	92	104 ± 4	pPECL-4	1577
Permease of the major facilitator superfamily						
PECL_1922	TAGGAGCGGATACAGTTGCT	TGCCCAGATACATAAGCCTG	140	95 ± 6	pPECL-4	1373
Type 2 lantibiotic biosynthesis protein LanM						
PECL_1943	ACACCAGAAGATCAGTATCTATCG	TCTTTGACACCAAATTGCGAC	178	105 ± 5	pPECL-6	3092
Hypothetical protein						
PECL_1947	CAGTTGCAGTTGAACCGTC	GCTAGTCATAGCGTCTCTCA	88	91 ± 6	pPECL-6	299
Putative ABC transporter, ATPase component						
bsrA [d]	GGAGGACTGGACCATCAG	CTCTCTTCGGTAGCCATCC	95	99 ± 15	chromosome	1937
Malonyl CoA-acyl carrier protein transacylase						
fabD	GACTGAAGGACGTTGCTCAC	CGATCTTTAATCAGGCGTAACC	105	106 ± 16	chromosome	923
Translation elongation factor G						
fusA	AACACATGATGGTGCTTCAC	TGATGCTTGACGCCAAACAG	246	94 ± 3	chromosome	2090
Guanylate kinase						
gmk [d]	AATGGCGAGGTTAATGGTG	CACATACTGTAGCGGTGTCC	131	100 ± 5	chromosome	614
ABC-type multidrug transporter						
horA [d]	GGATCATCAACTCAATCGGTC	CCAAAGTGTTGTTCGCAGC	155	94 ± 4	pPECL-8	1751
Isoleucyl-tRNA-synthetase						
ileS [c]	GGTCATGGTCTTCAGAATCAG	GGTTGAACAACGGCATAGTC	213	104 ± 2	chromosome	2785
ATP-dependent DNA helicase						
pcrA	ATGAGAAGATTGTTGCTGAGG	ACATCGTTACTAATTGGTATTGAGC	209	111 ± 12	chromosome	2264
6-phosphofructokinase						
pfkA	CTTAGTTGCTGGTGACATCC	TGATATGAACCATCGCCACC	191	105 ± 5	chromosome	971
Pyrroline-5-carboxylate reductase						
proC	TTAGTGTCGCTTGTCTCAGG	GAGAACTTCCTGCAAGAGCTG	252	95 ± 6	chromosome	791
Recombinase						
recA	GATCATTGGCACTTGATGAGG	CTCAGCAACAGCATGTAGTG	118	107 ± 19	chromosome	1052
RNA polymerase						
rpoB	GCTTCGTGAGATGTTCAACG	TCGCCAGTTTCGTGGTTGG	182	97 ± 3	chromosome	3605
pPECL-1: plasmid replication protein						
PECL_2013	GGACACCTGATGATGTCATACG	GATACTCCGATCACCTAGAAGC	136	96 ± 5	pPECL-1	692
pPECL-2: plasmid replication protein						
PECL_2011	AGAGGCAGTTGTGAGAGAGC	GAGCCGTCATGTTCGTTCAC	116	94 ± 5	pPECL-2	953
pPECL-3: glutathione reductase						
PECL_1987	TGGTCATGCCACGTTCAAGG	ACTATTGTGGGCGAGCTTGG	111	86 ± 3	pPECL-3	1334
pPECL-5: hypothetical protein						
PECL_1883	AGCGGTGATCGTGGTGATAC	GTTGTGACTGATGACGTTATGC	171	99 ± 7	pPECL-5	368

[a] Genes listed in the first section were chosen for RNA-seq verification; second section are for analysis of plasmid pPECL-4 and pPECL-6 gene expression; third section are genes that were analyzed in Bergsveinson *et al.* 2012 [11], but were included in this study for RNA-seq comparison; fourth section are primers that were used for plasmid copy number analysis.
[b] Amplification efficiency ± standard deviation.
[c] Also used as an inter-run calibrator for plasmid copy number analysis.
[d] Also used for plasmid copy number analysis as chromosome representative (*bsrA, gmk*) or for pPECL-8.
