# Supplementary material for: Transcriptome Sequence and Plasmid Copy Number Analysis of the Brewery Isolate Pediococcus claussenii ATCC BAA-344T during Growth in Beer
Source: PLoS One. 2013 Sep 6;8(9):e73627. doi: 10.1371/journal.pone.0073627 (PMC3765258; doi:10.1371/journal.pone.0073627)
Supplement: Table S2 — Genes added to the P . claussenii ATCC BAA-344T genome annotation. (PDF) [file pone.0073627.s003.pdf]

Table S2. Genes added to the *P. claussenii* ATCC BAA-344[T] genome annotation

Locus_tag	Gene Description	Location	Gene Start	Gene End	Strand	Fold Change [a]
PECL_2059	putative ncRNA between PECL_171 & PECL_172	chromosome	175194	175817	+	32.51
PECL_2057	bacterial small signal recognition particle RNA	chromosome	499951	500037	+	1.97
PECL_2045	membrane protein	chromosome	533959	534318	+	2.61
PECL_2056	transfer messenger RNA *ssrA*	chromosome	590129	590500	+	11.38
PECL_2046	hypothetical protein	chromosome	603906	604151	+	0.60
PECL_2060	putative ncRNA between PECL_686 & PECL_687	chromosome	698797	698985	+	63.91
PECL_2047	hypothetical protein	chromosome	799027	799155	-	0.97
PECL_2048	hypothetical protein	chromosome	863743	863970	-	1.66
PECL_2049	Prophage Lp1 protein 7, nonsense mutations	chromosome	879505	879915	-	0.63
PECL_2050	hypothetical protein	chromosome	966418	966747	-	0.89
PECL_2058	Ribonuclease P (RNase P) class B	chromosome	1016600	1016967	+	3.23
PECL_2051	50S ribosomal protein L33	chromosome	1099087	1099236	-	0.53
PECL_2052	vanZ like family protein	chromosome	1222725	1223096	+	0.94
PECL_2061	putative ncRNA between PECL_1459 & PECL_1460	chromosome	1410556	1410791	+	0.28
PECL_2062	putative ncRNA between PECL_1482 & PECL_1483	chromosome	1435477	1435632	+	0.66
PECL_2053	hypothetical protein	chromosome	1527892	1528242	-	2.83
PECL_2054	hypothetical protein	chromosome	1540183	1540503	+	2.20
PECL_2055	hypothetical protein	chromosome	1584901	1585119	+	1.00
PECL_2063	putative ncRNA between PECL_1907 & PECL_1867	pPECL-5	36037	36328	+	2.53
PECL_2064	putative ncRNA between PECL_2033 & PECL_2044	pPECL-8	4	375	+	9.97
PECL_2065	putative ncRNA between PECL_2033 & PECL_2034	pPECL-8	2152	2430	+	6.94

[a] Fold change indicates transcript levels in beer compared to those in MRS-B. Green font indicates significantly higher transcript levels in beer, whereas orange shows significantly higher levels in MRS-B (determined by *DESeq*, *p*-value < 0.01).
